# Serum magnesium and stable asthma: Is there a link?

**DOI:** 10.4103/0970-2113.71944

**Published:** 2010

**Authors:** Sibes Kumar Das, Arup Kumar Haldar, Indranath Ghosh, Samirendra Kumar Saha, Anirban Das, Saurabh Biswas

**Affiliations:** *Department of Respiratory Medicine, North Bengal Medical College, Susruta Nagar, Darjeeling, India*; 1 *Department of Critical Care Medicine, Columbia Asia Hospital, Kolkata, India*; 2 *Department of Respiratory Medicine, Calcutta National Medical College, Kolkata, India*; 3 *Department of Respiratory Medicine, Calcutta National Medical College, Kolkata, India*

**Keywords:** Link, serum magnesium, stable asthma

## Abstract

**Background:**

Although magnesium is used through intravenous and inhalation route in the management of asthma, actual prevalence of hypomagnesemia in asthma is not known. We conducted this study: 1) to detect the prevalence of hypomagnesemia in stable asthma and 2) to assess the significance of hypomagnesemia in these patients.

**Design:**

Prospective clinical study.

**Setting:**

Department of Respiratory Medicine, Calcutta National Medical College, Kolkata.

**Period of Study:**

Four months from January, 2007, to April, 2007.

**Materials and Methods:**

Fifty patients attending outpatients department of respiratory medicine with stable asthma were randomly selected. They were assessed clinically and their serum magnesium levels were measured. This was compared with the serum magnesium values of 45 nonasthmatic healthy controls.

**Results:**

Out of 50 patients, 14 had hypomagnesemia. Possible relationship of hypomagnesemia with tachycardia, tachypnoea, severity of asthma, medication use, and previous and future exacerbations were analyzed.

**Conclusion:**

There was statistically significant association of hypomagnesemia with tachypnoea, severe asthma, use of long-acting β-agonist, inhaled corticosteroids, theophylline, use of ≥ 3 medications, previous and future exacerbations but not with tachycardia or use of short-acting β_2_ -agonist or montelukast.

## INTRODUCTION

Magnesium (Mg^++^) is the fourth most common cation in the body and the major intracellular divalent cation. Mg^++^ in extracellular fluid is crucial for normal neuromuscular activities; intracellular Mg^++^ forms a key complex with ATP and is an important cofactor for a wide range of enzymes, transporters, and nucleic acids needed for normal cellular function, replication, and energy metabolism.[1] Total body Mg^++^ is about 25 g (1000 mmol). About 50% of it is in the bones, only 1% is in the extracellular fluid, and the rest is within the cells. About 30% of the serum Mg^++^ is bound to albumin. Mg^++^ has several actions on rabbit bronchial airways including relaxation of airway smooth muscle, bronchodilation, anticholinergic effects, and stabilization of mast cells.[2] Magnesium deficiency has been shown to correlate with a number of chronic diseases like hypertension, diabetes mellitus, and hyperlipidemia.[3] Mg^++^ is proved to be useful in the management of acute coronary syndrome,[4] Pre-eclampsia and eclampsia,[5] and ventricular tachyarrythmia.[6] Both intravenous and inhaled magnesium have been shown to be beneficial in acute attack of asthma.[7,8] But the prevalence of hypomagnesemia in stable asthma and its impact on asthma is not well studied. The present study was done to evaluate the levels of serum magnesium in patients with stable asthma, to assess the prevalence of hypomagnesemia among them and the possible significance of hypomagnesemia in these patients.

## MATERIALS AND METHODS

Patients with stable asthma registered between January, 2007, to April, 2007 at outpatients department of respiratory medicine, Calcutta National Medical College, Kolkata, a tertiary care hospital and a teaching institution, were included in this study. Written informed consent was obtained from each patient.

The inclusion criteria were as follows:

- Stable asthma (defined as no history of exacerbation at the time of presentation or within last 1 week).
- Age more than 12 years.
- Nonsmoker.

The exclusion criteria were as follows:

- Medical disorders like chronic kidney disease, diabetes mellitus, alcoholism, diarrhea.
- Patients on diuretic therapy.
- Pregnancy.
- Intermittent asthma.
- Cardiac disease.

All the patients were evaluated with detailed history, general survey, and examination of the respiratory system. The diagnosis of asthma was confirmed by the presence of any of the following:

- Previous document of spirometric evidence of asthma.
- Spirometric evidence of asthma at the time of presentation.

FEV_1_ < 80% of predicted, FVC < 80% of predicted, FEV_1_/FVC < 70%, and improvement of FEV_1_ by >200 ml and 12% after 400 *μ*g of salbutamol inhalation through metered dose inhaler with spacer was considered spirometric evidence of asthma.

Severity of asthma was assessed on the basis of GINA guidelines.[9]

Two milliliter of fasting venous blood was drawn from each patient for estimation of serum magnesium with autoanalyzer method using Erba CHEM-5 Plus V_2_ autoanalyzer machine (Erba Diagnostics Mannheim GmbH, Germany). Serum albumin level was also estimated in all patients to exclude hypoalbuminemia as a cause of hypomagnesemia. Each patient was also questioned regarding their inhaled and oral medications and the presence of any history of acute exacerbation in the previous 3 months. Acute exacerbation of asthma was defined as per GINA guideline,[9] i.e., episode of progressive increase in dyspnoea, cough, wheezing, or chest tightness, associated with a fall in PEF or FEV_1_. They were also followed up for the next 3 months for the development of any acute exacerbation. The relationship of hypomagnesemia with tachycardia (heart rate > 100 beats/min), tachypnoea (respiratory rate>20 breaths/min), severity of asthma, types of medication use, total number of medications used, occurrence of acute exacerbation in past 3 months, and in the next 3 months follow up were assessed and their statistical significance was calculated using Chi-square test and Fisher’s test.

## RESULTS

Total 50 patients of stable asthma who attended our outpatients department and satisfied the inclusion criteria were enrolled for the study. There were 18 (36%) males and 32 (64%) females. The age and sex distribution of the patients are shown in Table 1.

**Table 1:** Distribution of patients according to age and sex

Age group (years)	Male(*n* = 18)		Female (*n* = 32)	
	Number	Percentage	Number	Percentage
13–20	5	27.7	9	28.1
21–30	3	16.6	8	25.0
31–40	3	16.6	6	18.7
41–50	1	5.5	5	15.6
51–60	2	11.1	2	6.2
>60	4	22.2	2	6.2

Age ranged from 13 years to 71 years (mean 42.7 years). Asthma in this study is found to be more common in female and mostly affects the younger age group (<30 years) in both sexes.

Assessment of severity of asthma in them revealed that 28 (56%) had mild persistent asthma, 12 (24%) had moderate persistent asthma, and 10 (20%) had severe persistent asthma.

Total 35 patients (70%) were using short-acting β_2_ -agonist (SABA) daily, 34 patients (68%) were using inhaled corticosteroid (ICS), 22 (44%) patients were using long-acting β_2_ -agonist (LABA), 11 patients (22%) were using montelukast (MTK), and 13 patients (26%) were using theophylline (THP). Out of 50 patients, 17 (34%) patients were using ≥ 3 medications for the control of asthma.

Total 10 patients (20%) gave history of acute exacerbation in past 3 months and 5 patients (10%) developed acute exacerbation in the 3 months follow up.

In this study, serum magnesium value between 1.8 and 3.0 mg/dl was considered normal and any value below 1.8 mg/dl was considered as hypomagnesemia. Using this cut-off value, 14 (28%) patients were found to have hypomagnesemia and their serum magnesium value ranged between 0.91 and 1.6 mg/dl. Rest 36 patients (72%) had normal serum magnesium level. Serum albumin level was measured in all patients and none of them had hypoalbuminemia.

Here 45 nonasthmatic healthy control persons were selected. The controls were subject to similar inclusion (except being nonasthmatics) and exclusion criteria. The age and sex distributions of the control are depicted in Table 2.

**Table 2:** Age and sex distributions of nonasthmatic controls

Age group (years)	Male(*n* = 20)		Female (*n* = 25)	
	Number	Percentage	Number	Percentage
13–20	5	25.0	3	12.0
21–30	4	20.0	6	24.0
31–40	2	10.0	7	28.0
41–50	3	15.0	2	8.0
51–60	2	10.0	3	12.0
>60	4	20.0	4	16.0

Serum magnesium and serum albumin level were measured to look for hypomagnesemia and hypoalbuminemia in them. All of them had serum magnesium values between 1.8 and 3 mg/dl, and serum albumin level between 3.5 and 5.5 g/dl.

The relationship of serum magnesium values with tachycardia, tachypnoea, severity of asthma, types of medications used, total number of medication use, and episode of exacerbation in previous 3 months and future 3 months were assessed. The results are presented in Table 3.

**Table 3:** Relationship of serum magnesium levels with different variables in asthma

Variables	Hypomagnesemia (*n* = 14)	Normomagnesemia (*n* = 36)	*P* value
Tachycardia			
Yes	4	4	0.196
No	10	32	
Tachypnoea			
Yes	8	7	0.023
No	6	29	
Severity of asthma			
Mild	4	24	0.025
Moderate to severe	10	12	
Total medication			
<3	5	28	0.007
≥3	9	8	
Use of SABA			
User	10	25	1
Nonuser	4	11	
Use of ICS			
User	13	21	0.021
Nonuser	1	15	
Use of LABA			
User	11	11	0.003
Nonuser	3	25	
Use of MTK			
User	5	6	0.252
Nonuser	9	30	
Use of THP			
User	7	6	0.04
Nonuser	7	30	
Past exacerbation			
Yes	6	4	0.019
No	8	32	
Exacerbation during follow-up			
Yes	4	1	0.018
No	10	35	

From this Table, it is evident that hypomagnesemia is related to tachypnoea, moderate to severe asthma, use of ICS, LABA and THP, use of ≥3 medications, past history of asthma exacerbation in previous 3 months, and also occurrence of future exacerbation in the 3 months follow-up. However, this study did not reveal any statistically significant relationship of hypomagnesemia with tachycardia, use of SABA, and montelukast.

## DISCUSSION

Magnesium is an intracellular ion. Serum Mg^++^ levels correlate poorly with the total body store and serum Mg^++^ may appear normal in spite of depletion of body store. The estimation of Mg^++^ level in RBC, WBC, or muscle cell will be more representative of the body store but it is expensive, not easily available and not clinically applicable. Serum Mg^++^ level, therefore, is often used to assess the change in the Mg^++^ status despite its limitation. We also have measured serum Mg^++^ level is our study as these expensive tests are not available in our institution.

Both intravenous and inhaled magnesium have been used for decades in the treatment of asthma during acute and chronic stable state.[7,8] International guideline has also recommended the use of intravenous magnesium sulfate in the treatment of acute severe asthma, especially if FEV_1_ is between 25–30% of predicted at presentation, or if there is poor response with SABA.[9] The possible mechanisms of action of magnesium on airways include inhibition of vascular and bronchial smooth muscle contraction, inhibition of acetylcholine release from cholinergic nerves, promotion of nitric oxide and prostacycline generation, and stabilization of smooth muscle.[2,10]

The prevalence of hypomagnesemia in asthma is variable. Although there are reports of significantly high prevalence of hypomagnesemia both in acute asthma and chronic stable asthma when compared with general nonasthmatic population,[11,12] these have not been confirmed by subsequent studies.[13,14] However, there are reports of low magnesium level in skeletal muscle[15] and polymorphonuclear leucocytes[16] in asthmatic patients.

Several studies have proved beyond doubt that hypomagnesemia in asthma is associated with increased incidence of wheeze, impairment of lung function, and more significantly, increased bronchial hyper-reactivity (BHR).[17,18] In another study,[19] it has been shown that there were high prevalence of hypomagnesemia (27%) in stable asthmatics, with significantly higher incidence of acute asthma attacks and hospitalization (40% vs 11.8%, *P* <0.01); serum magnesium level also correlated significantly with severity of asthma (*P*<0.04). These findings are similar to our study which has shown high prevalence of hypomagnesemia (28%) and the significant association of hypomagnesaemia with severe asthma and previous and future exacerbations. Therapeutic and dietary supplementation of magnesium have been shown to either reduce the BHR[8,18] or improve the symptom score of asthma without objective improvement in BHR.[20] It is probable that increase in the severity of asthma or increased frequency of exacerbation seen in asthmatics with hypomagnesemia is related to an increase in the BHR produced by magnesium deficiency. Several mechanisms have been put forward to explain this increase in BHR, like increased production of acetylcholine in cholinergic nerve endings,[21] increased histamine release from mast cell,[10] increase in Ca^++^ flux into airway smooth muscle cell,[15] or increased production of interleukin 1, interleukin 6.[22]

Causes of magnesium deficiency in asthma may be multifactorial. It may be genetically determined.[23] It is also attributed to either low magnesium intake in asthmatic or increased urinary loss of magnesium as a side-effect of therapy with ß_2_ -agonist, corticosteroid, and theophylline.[12,24,25] But hypomagnesemia is found to be present in the patients who are not on treatment with these drugs or even after withdrawal of the drugs.[15,19,20] In our study we, however did not consider the dietary pattern of the patients but found hypomagnesemia to be more common with increased number of antiasthma medications like LABA, ICS, and THP.

## CONCLUSION

It can be concluded from the present study that hypomagnesemia is more prevalent in stable asthmatics than nonasthmatic control. There is statistically significant correlation of hypomagnesemia with tachypnoea, severe asthma, use of LABA, ICS and THP, requirement of multiple medications (≥3), and the presence of exacerbation in past 3 months and in next 3 months follow-up. This study could not find any significant correlation between hypomagnesemia and the presence of tachycardia or use of SABA and MTK.

There are few pitfalls in our study. We measured serum concentration of magnesium which may not be representative of actual body store. We did not consider the amount of dietary magnesium in the patients. So, further studies using large number of patients, and measuring Mg^++^ level in RBC, WBC, or muscle cell in addition to serum Mg^++^ will give better insight in this field. The cause of hypomagnesemia in asthmatics and whether magnesium supplementation has a role in asthmatic population are important fields of further research.
